# Effects and mechanisms of apolipoprotein A-V on the regulation of lipid accumulation in cardiomyocytes

**DOI:** 10.1186/s12944-018-0692-x

**Published:** 2018-03-12

**Authors:** Jun Luo, Li Xu, Jiang Li, Shuiping Zhao

**Affiliations:** 1 Department of Cardiology, The Second Xiangya Hospital, Central South University, Changsha, Hunan 410011 China; 2 Department of The Second Chest Medicine, The Affiliated Cancer Hospital of Xiangya School of medicine, Central South University, Changsha, Hunan 410013 China

**Keywords:** Apolipoprotein A-V, HL-1 cells, Triglyceride, Lipid droplets

## Abstract

**Background:**

Apolipoprotein (apo) A-V is a key regulator of triglyceride (TG) metabolism. We investigated effects of apoA-V on lipid metabolism in cardiomyocytes in this study.

**Methods:**

We first examined whether apoA-V can be taken up by cardiomyocytes and whether low density lipoprotein receptor family members participate in this process. Next, triglyceride (TG) content and lipid droplet changes were detected at different concentrations of apoA-V in normal and lipid-accumulation cells in normal and obese animals. Finally, we tested the levels of fatty acids (FAs) taken up into cardiomyocytes and lipid secretion through [^14^C]-oleic acid.

**Results:**

Our results show that heart tissue has apoA-V protein, and apoA-V is taken up by cardiomyocytes. When HL-1 cells were transfected with low density lipoprotein receptor (LDLR)-related protein 1(LRP1) siRNA, apoA-V intake decreased by 53% (*P*<0.05), while a 37% lipid accumulation in HL-1 cells remain unchanged. ApoA-V localized to the cytoplasm and was associated with lipid droplets in HL-1 cells. A 1200 and 1800 ng/mL apoA-V intervention decreased TG content by 28% and 45% in HL-1 cells, respectively and decreased TG content by 39% in mouse heart tissue (*P*<0.05). However, apoA-V had no effects on TG content in either normal HL-1 cells or mice. The levels of FAs taken up into cardiomyocytes decreased by 43% (*P* < 0.05), and the levels of TG and cholesterol ester secretion increased by 1.2-fold and 1.6-fold, respectively (*P* < 0.05).

**Conclusion:**

ApoA-V is a novel regulator of lipid metabolism in cardiomyocytes.

## Background

Apolipoprotein AV (ApoA-V) is a member of the apoA class of proteins, which has been shown to be a key regulator of plasma triglyceride (TG). Pennacchio et al. found that mice expressing a human apoA-V transgene exhibited a 65% decrease in plasma TG concentration compared with control mice [1]. The adenovirus-mediated expression of apoA-V in mice resulted in a decrease in both plasma TG and total cholesterol by 70 and 40%, respectively [2]. Recently, Zheng et al. found that apoA-V is taken up by human adipocytes and decreases cellular TG content [3, 4]. These results strongly indicate an inverse relationship between apoA-V and TG levels. Hence, a lack of normal functioning apoA-V is a risk factor for hypertriglyceridemia. However, Ress et al. established a cell culture model of apoA-V knockdown and found that apoA-V suppression resulted in a significant decrease of intracellular TG levels [5]. These findings were consistent with another experiment from Shu et al., who reported a significant increase in TG content in the livers of apoA-V transgenic mice [6]. These results suggest that apoA-V not only has known extracellular effects but also exerts intrahepatic effects, resulting in increased lipid accumulation. However, the exact underlying mechanisms of how apoA-V function affects other cells, such as cardiomyocytes, are not fully understood.

A healthy adult heart derives most of its energy from mitochondrial oxidation of FAs [7, 8]. In cardiomyocytes, FAs are either directly delivered to mitochondria and subsequently oxidized or esterified to TG for temporary storage in cytoplasmic lipid droplets [7, 8]. The synthesis and hydrolysis of myocardial TG stores is a highly dynamic process that is tightly controlled, contributing to normal cardiac metabolism and function. Additionally, it is well established that obesity greatly increases the risk for coronary artery disease [9] and heart failure [10]. In addition, excessive myocardial TG accumulation is associated with impaired heart function [11], highlighting the crucial role that TG metabolism plays in the regulation of normal cardiac metabolism and function. As mentioned above, apoA-V has been demonstrated to play a critical role in TG metabolism and energy balance; however, until now, the exact physiologic effects and underlying mechanisms of apoA-V have been unclear. Whether apoA-V can regulate FA and TG metabolism in cardiomyocytes remains to be fully explored.

Thus, the aim of this study was to investigate the physiological effects of apoA-V on cardiomyocytes with lipid accumulation. We hypothesized that apoA-V can be taken up by cardiomyocytes and exerts its effects on cardiac lipid metabolism. We thus investigated the effects of apoA-V overexpression in an adenovirus-mediated obese mouse model in vivo*.* Furthermore, we established an in vitro cell culture model of lipid accumulation to study its role in intracellular lipid metabolism.

## Methods

### Experimental animals

C57BL/6 male mice (23 ± 1 g) with conventional microbiological status (Animal Experiment Center of Central South University, *n* = 24) were acclimatized for 14 days prior to experiments (12:12-h light/dark photoperiod, 23 °C, 55–60% humidity, and tap water ad libitum). All mice were fed a high-fat diet (HFD) containing 59% fat, 11% protein and 30% carbohydrate for 8 weeks [12]. All research involving these animals was approved by a local University ethics review board and conformed to the Home Office Guidance on the operation of Animals (Scientific Procedures) Act-1986, the institutional guidelines, and the Directive 2010/63/EU of the European Parliament.

### Adenoviral expression of ApoA-V in C57BL/6 mice

The construction of a recombinant replication-deficient adenoviral vector expressing human apoA-V (Ad-hapoA-V) is previously described [2]. A range of 0–5 × 10^8^ plaque-forming units (pfu) of Ad-apoA-V (total viral dose was adjusted to 5 × 10^8^ with empty vector (Ad-mock)) was injected into the tail vein of HFD mice (*n* = 6/group) 3 h after injection of Ad-LacZ (5 × 10^8^ pfu) [13]. Seven days after injection, mice were sacrificed via a lethal overdose of pentobarbital (i.p.), at which time hearts were removed and perfused via the aorta with chilled buffer, and papillary muscles including part of the septum and septal artery were dissected from the right ventricle [14]. Expression levels of apoA-V were determined by real-time PCR (TaqMan, Invitrogen; Thermo Fisher Scientific, Inc.). Western blot analysis of heart samples was used to estimate apoA-V expression [2].

### Cell culture and treatments

HL-1 cells (from the AT-1 mouse atrial cardiomyocyte tumour lineage) were cultured and seeded onto gelatine/fibronectin-coated plastic cluster plates or flasks (BD Biosciences, USA) at a density of 5 × 10^5^ cells/well and cultured in Claycomb medium with supplements at 37 °C and 5% CO_2_ [15]. Recombinant human apoA-V was produced in *Escherichia coli* and isolated as described by Beckstead et al. [16]. The effect of apoA-V intervention on lipid accumulation was tested in HL-1 cells incubated for 24 h in Claycomb medium supplemented with 1% foetal bovine serum (FBS) (Lonza, Switzerland) in the presence of 200 μM low endotoxin fatty acid free BSA ± 500 μM conjugated sodium oleate (Nu-Chek-Prep, USA). ApoA-V was given at different concentrations between 50 to 1800 nM to test the maximum effect of apoA-V on TG content. Target TG regulation was observed at an apoA-V concentration of 1800 nM; hence, 1800 nM apoA-V was used in all subsequent experiments.

### siRNA transfection

HL-1 cells with normal lipid accumulation were transfected with small interfering RNA (siRNA) targeting low density lipoprotein receptor-related protein 1 (LRP1) (LRP1 NM_008512 Validated siRNA, GENECHEM, China) or non-silencing control siRNA (GENECHEM, China) using the lipotransfection method (HiPerfect, Qiagen, Hilden, Germany). Transfected cells were maintained in cell culture transfection media containing 1 nM to 100 nM siRNA and HiPerfect Transfection reagent for up to 48 h. LRP1 silencing by siRNA was quantified by Western blotting and fluorescence-based real-time PCR. All siRNA transfection experiments were performed in duplicate. The number of independent experiments performed in each setting is shown in the results section.

### Pulse-chase experiment

For endocytosis studies, apoA-V was labeled with [^125^I] Iodogen (Sigma-Aldrich, USA) [17]. HL-1 cells were incubated (pulsed) with [^125^I]-labeled apoA-V (320 ng; ∼2 × 106 cpm/well) in serum-free medium for 2 h at 37 °C. After incubation, cells were washed three times with ice-cold PBS, treated with heparin (10 mg/mL) and then chased at 37 °C in fresh medium for various times (up to 24 h). Cells and media were collected for each chase period and analysed for the presence of [^125^I]-labeled apoA-V. Radioactivity (cpm) was normalized to cell protein concentrations, and data are expressed as the percentage of total cpm/mg protein at each time point.

To investigate whether LDLR family members participate in the endocytosis of apoA-V, HL-1 cells were first transfected with LRP1 siRNA and subjected to pulse-chase experiment.

### Immunofluorescence and confocal microscopy

HL-1 cells were co-incubated with 0.5 mM oleic acid and 1800 nM apoA-V (24 h), washed three times with PBS, fixed with 4% paraformaldehyde, incubated with rabbit anti-apoA-V (1:200 dilution) overnight at 4 °C, and incubated with goat anti-rabbit Alexa 594 secondary antibody. Localization of apoA-V on lipid droplets in cardiomyocytes were visualized by using Oil Red O staining.

### Measurement of long chain fatty acid uptake and lipid secretion

The effects of apoA-V addition on FA uptake was assessed by incubating HL-1 cells with apoA-V (180 ng/mL) and oleic acid/BSA (0.4 mM) for 24 h followed by ^14^C-labeled oleic acid (Amersham Life Science Little Chalfont, UK; 0.5 μCi per well; 0.5 mM final concentration) [18]. After 10 min, uptake was terminated, and unbound substrate was removed by washing the cells with ice-cold depletion medium containing 0.2 mmol/L phloretin, and incorporated radioactivity was measured with a scintillation counter. To assess the effects of apoA-V on secretion of TG and cholesterol ester, HL-1 cells were co-incubated with apoA-V (1800 ng/mL) and ^14^C-oleic acid/BSA (0.5 μCi per well; final concentration 0.5 mM) for 24 h. Total lipid content in the cells and the medium was extracted and purified using the method described by Bligh and Dyer [19]. Neutral lipid subclasses were separated by thin-layer chromatography precoated silica gel G with a solution mixture of petroleum ether, diethylether, and acetic acid (80:20:1, by vol.). The radioactivity of TG and cholesterol ester were measured by scintillation counting.

### Immunohistochemistry and oil red O staining

Expression of apoA-V in heart tissue was detected immunohistochemically using a rabbit polyclonal antibody (ab71265 Abcam, USA) and the streptavidin–biotin peroxidase technique. Cardiac and liver lipid accumulation was determined by Oil Red O staining (Sigma Aldrich).

### Measurement of HL-1 cells and heart TG content

Lipid extracts of HL-1 cells and heart tissue were assayed for TG using a Triglyceride Quantification Kit (BioVision, USA).

### Total RNA preparation and real-time quantitative PCR analysis

Total RNA was extracted from HL-1 cells and heart tissue using the RNeasy kit (Qiagen). One μg of RNA was reverse-transcribed to cDNA using the QuantiTect RT kit (Qiagen) per manufacturer’s protocol. The abundance of transcripts was assessed by real-time PCR on a 7500 Fast Real-Time PCR system (Applied Biosystems) with a SYBR Green detection system. Oligonucleotide primers are listed in Table 1. β-actin was used as housekeeping gene, and data were normalized for the efficiency of amplification, as determined by a standard curve included on each run.

**Table 1:** Primer sequence

Target	Species	Sequence Table 1 Primer sequences
PPARα	mouse	Forward 5’- CCT CAG GGT ACC ACT ACG GAG T-3’
		Reverse 5’-GCC GAA TAG TTC GCC GAA-3’
PPARγ	mouse	Forward 5’-AGG CCG AGA AGG AGA AGC TGT TG-3’
		Reverse 5’-TGG CCA CCT CTT TGC TGT GCT C-3
DGAT1	mouse	Forward 5’- TCC GCC TCT GGG CAT TC -3’
		Reverse 5’- GAA TCG GCC CAC AAT CCA − 3’
DGAT2	mouse	Forward 5’- TGG AAC ACG CCC AAG AAA G − 3’
		Reverse 5’- CAC ACG GCC CAG TTT CG − 3’
SCD	mouse	Forward 5’-TTC TTA CAC GAC CAC CAC CA-3’
		Reverse 5’-CCG AAG AGG CAG GTG TAG AG-3’
GPAT	mouse	Forward 5’-TCA CAA GGG TCA ACT CGA GAT G-3’
		Reverse 5’-GTG CAC CGG CAG AAA CAA G-3’
ATGL	mouse	Forward 5’- AGG ACA GCT CCA CCA ACA TC-3’
		Reverse 5’- TGG TTC AGT AGG CCA TTC CT-3’
HSL	mouse	Forward 5’-GCT TGG TTC AAC TGG AGA GC-3’
		Reverse 5’-GCC TAG TGC CTT CTG GTC TG − 3’
FAT/CD36	mouse	Forward 5’- GAA CCT ATT GAA GGC TTA CATCC -3’
		Reverse 5’- CCC AGT CAC TTG TGT TTT GAA C -3’
FABP	mouse	Forward 5’- CCG CAG ACG ACA GGA-3’
		Reverse 5’- CTC ATG CCC TTT CAT AAA CT-3’
FATP	mouse	Forward 5’- AAA AGG AGC TGC CTC TG-3’
		Reverse 5’- AAG GAG CCT ATC AGA AAC C-3’
PGC-1α	mouse	Forward 5’- CCC TGC CAT TGT TAA GAC -3’
		Reverse 5’- GC TGC TGT TCC TGT TTT C -3’
MCAD	mouse	Forward 5’-TGGCATATGGGTGTACAGGG-3’
		Reverse 5’-CCAAATACTTCTTCTTCTGTTGATCA-3’
LCAD	mouse	Forward 5’-GGAGTAAGAACGAACGCCAA-3’
		Reverse 5’-GCCACGACGATCACGAGAT-3’
CPT-1	mouse	Forward 5’-ACT CCT GGA AGA AGA AGT TCA-3’
		Reverse 5’-AGT ATC TTT GAC AGC TGG GAC-3’
GAPDH	mouse	Forward 5’- ACC CAG AAG ACT GTG GAT GG-3’
		Reverse 5’- ACA CAT TGG GGG TAG GAA CA-3’

### Western blot analysis

Heart tissue and total cell lysates were prepared with a lysis buffer containing 50 mM Tris (pH 7.4), 150 mM NaCl, 1 mM EDTA, 1% Triton X-100, 0.5% sodium deoxycholate, and a protease inhibitor. Plasma sample was subjected to 12% SDS-PAGE, and proteins were transferred to PVDF membranes. The ApoA-V protein was detected by rabbit polyclonal antibody specific to ApoA-V (primary antibody) and HRP conjugated goat anti rabbit IgG (secondary antibody). Immunoblot signals were visualized using ECL detection reagents and quantified by densitometry analysis.

### Statistical analyses

Results are presented as the mean ± SEM or SD. Group comparison were made using Student’s t-test or ANOVA. *p* < 0.05 were considered statistically significant.

## Results

### Heart tissue from both normal and obese mice contain apoA-V

**Fig. 1:**
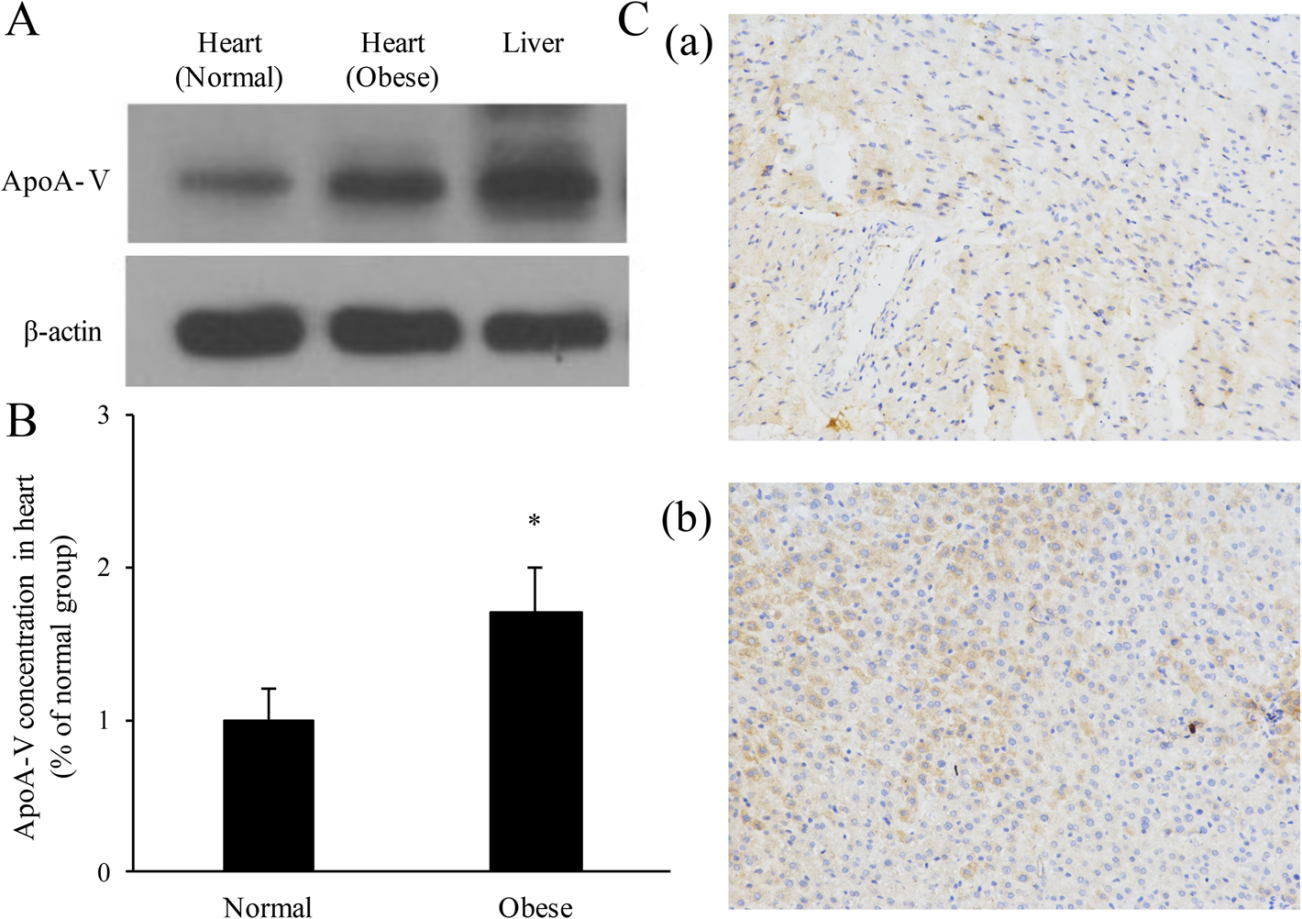
Expression of apoA-V in both normal and obese mice. Normal and obese mice were sacrificed, and the heart tissue was rapidly excised. Western blot (**A** and **B**) and immunohistochemistry (**C**) results show that heart tissues from both groups contained apoA-V. Compared to normal mice, in the heart tissue of obese mice, the amount of apoA-V was increased. Liver tissues were used as a positive control. (**C**-a: immunohistochemistry image of heart tissue; **C**-b: immunohistochemistry images of liver tissue)

ApoA-V was expressed in the liver and intestine but was not expressed in the heart tissue [1, 20]. However, cardiomyocytes can take up apoA-V from the blood, so apoA-V should be found in heart tissue. To test this hypothesis, we sacrificed normal and obese mice and rapidly excised the hearts. Western blot (Fig. 1A and B) and immunohistochemistry (Fig. 1C) results showed that both normal and obese mice contain apoA-V in their heart tissue. Compared to normal mice, obese mice exhibited increased levels of apoA-V. Liver tissues were used as a positive control. These results demonstrate that apoA-V can be taken up by cardiomyocytes.

### ApoA-V is taken up into cardiomyocytes in part via binding to members of the LDLR family

**Fig. 2:**
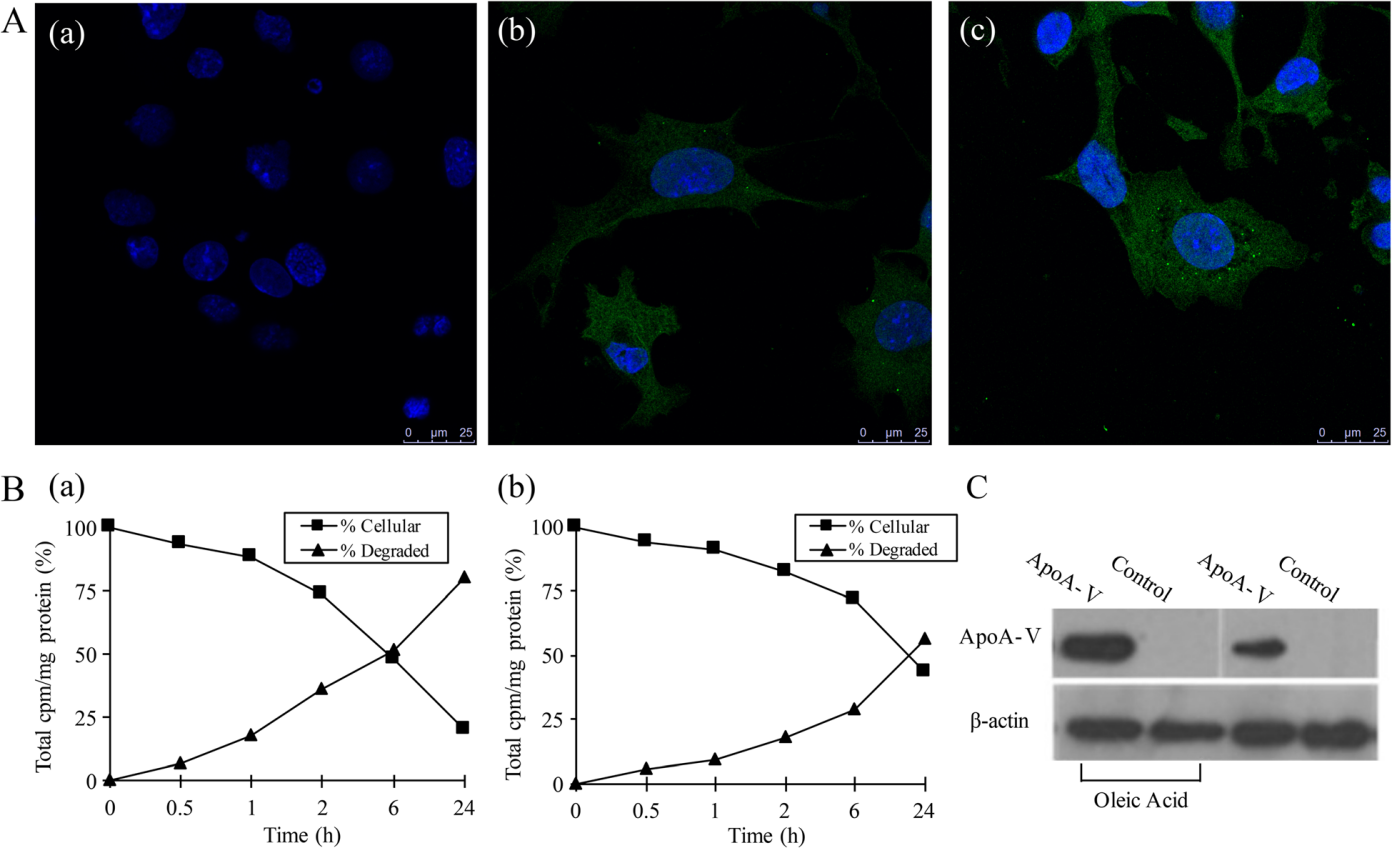
Endocytosis of apoA-V in both normal and lipid-accumulated HL-1 cells. **A** Subcellular localization of apoA-V detected by fluorescence and confocal microscopy. ApoA-V was localized within HL-1 cells with (**A**-c) or without (**A**-b) lipid accumulation (scale bar: 25 μm; nuclei: blue; apoA-V: green; A-a: normal HL-1 cells without apoA-V). **B** The [^125^I] radioactivity detection results of [^125^I]-apoA-V in HL-1 cells. In normal HL-1 cells, ~ 20% of [^125^I]-apoA-V uptake remained intracellular over a 24 h chase period, while 80% was degraded (**B**-a); however, in HL-1 cells with lipid accumulation, ~ 44% of [^125^I]-apoA-V uptake remained intracellular, and 56% was degraded over a 24 h chase period (**B**-b). **C** Western blot analysis of the uptake of apoA-V by HL-1 cells. β-actin served as a loading control

To investigate whether apoA-V can be taken up into cardiomyocytes and whether apoA-V uptake and its degradation are different between normal lipid accumulating cardiomyocytes, the ^125^I test was applied. Confocal fluorescence microscopy images (Fig. 2A) show that compared to normal HL-1 cells without apoA-V (Fig. 2A-a), apoA-V is taken up into HL-1 cells with (Fig. 2A-c) or without (Fig. 2A-b) lipid accumulation. The radioactivity detection results (Fig. 2B) showed that in normal HL-1 cells (Fig. 2B-a), ~ 20% of [^125^I]-apoA-V uptake remained intracellular over a 24 h chase period, while 80% was degraded; however, in HL-1 cells with lipid accumulation (Fig. 2B-b), ~ 44% of [^125^I]-apoA-V uptake remained intracellular, while 56% was degraded over a 24 h chase period. Furthermore, both normal and lipid-accumulated HL-1 cells were incubated for 4 h with 200 ng/mL apoA-V and subjected to Western blot analysis (Fig. 2C), which showed that apoA-V was detected in these HL-1 cells after 4 h of incubation. Together, these results demonstrate that apoA-V is taken up by HL-1 cells, and apoA-V is degraded more slowly in cases of lipid accumulation.

**Fig. 3:**
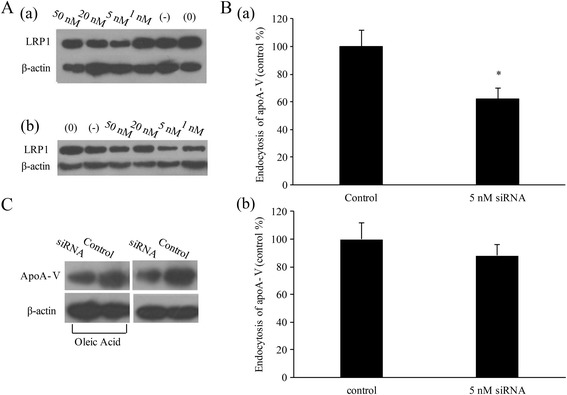
LDLR family members participate in endocytosis of apoA-V in HL-1 cells. **A** Determination of the optimum transfection concentration of LRP1 siRNA by Western blot analysis. **B** Radioactivity changes of [^125^I]-apoA-V in HL-1 cells after transfection of LRP1 siRNA. Data are shown as the mean ± SE of values from three independent experiments. **C** Western blot analysis of the effect of LRP1 on the endocytosis of apoA-V by HL-1 cells. β-actin served as a loading control

To further investigate whether LDLR family members participate in the endocytosis of apoA-V, both normal and lipid-accumulated HL-1 cells were transfected with LRP1 siRNA. First, we determined the best transfection concentration of LRP1 siRNA (5 nmol/L, Fig. 3A). After transfection of LRP1 siRNA in normal HL-1 cells, [^125^I]-apoA-V uptake was significantly decreased by 53% (*p* < 0.05, Fig. 3B-a) and 37% in lipid-accumulated HL-1 cells (Fig. 3B-b, no significant difference). To confirm these data, Western blot analysis (Fig. 3C) also illustrated that pre-incubation with LRP1 siRNA significantly inhibits levels of apoA-V taken up into normal HL-1 cells. However, there is no significant difference in HL-1 cells with lipid accumulation.

### ApoA-V significantly decreases TG content and the number of lipid droplets in HL-1 cells

**Fig. 4:**
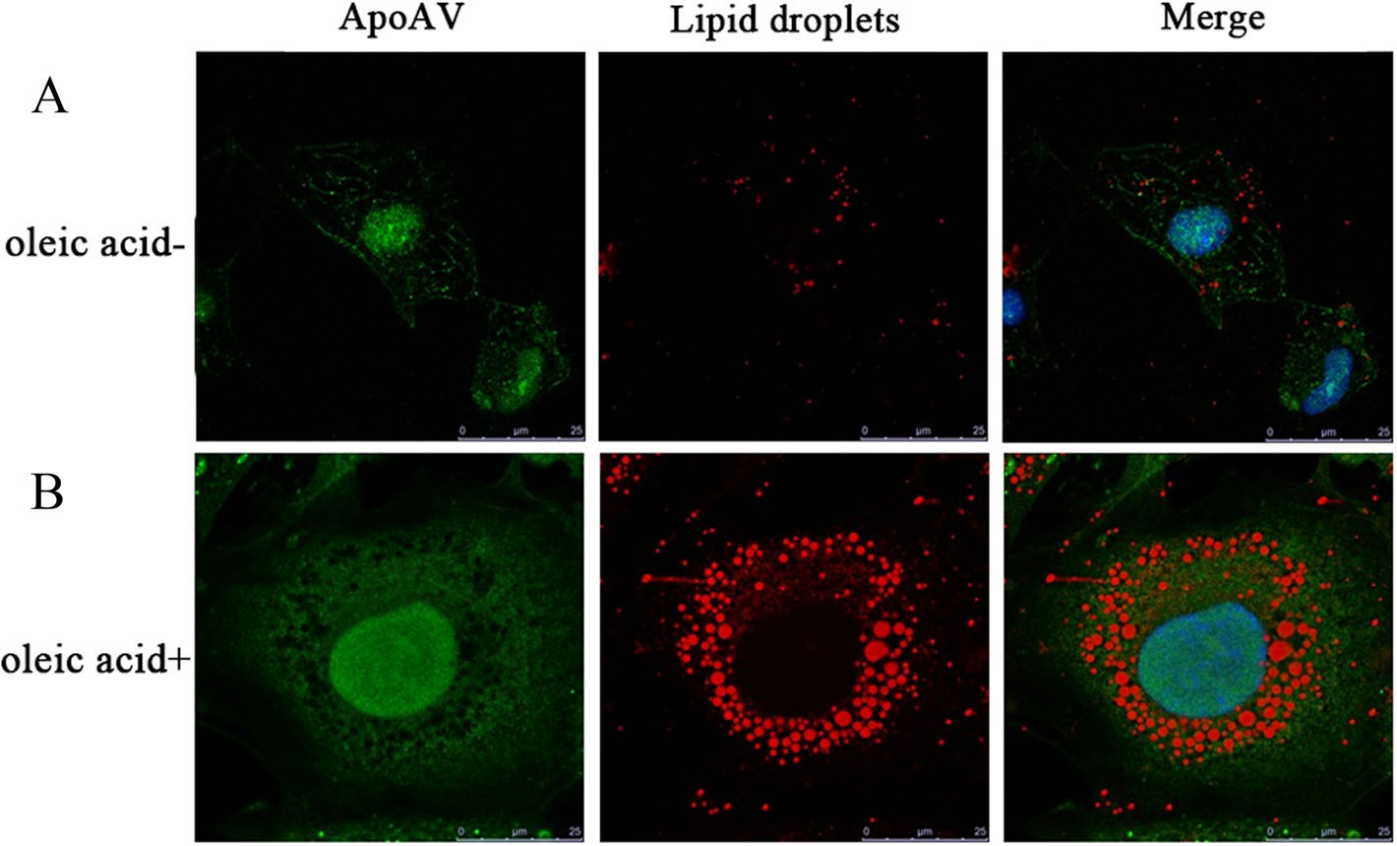
Association of apoA-V and lipid droplets in HL-1 cells. HL-1 cells were incubated with (**b**) or without (**a**) 0.5 mM oleic acid for 24 h. (scale bar 25 μm; lipid droplets: red; nuclei: blue; apoA-V: green)

To investigate the localization of apoA-V in the cytoplasm, HL-1 cells were incubated with 0.5 mM oleic acid for 24 h followed by incubation with 600 ng/mL apoA-V for 1 h. Confocal microscopy images (Fig. 4a and b) showed that apoA-V was localized to the cytoplasm and was associated with lipid droplets in HL-1 cells.

**Fig. 5:**
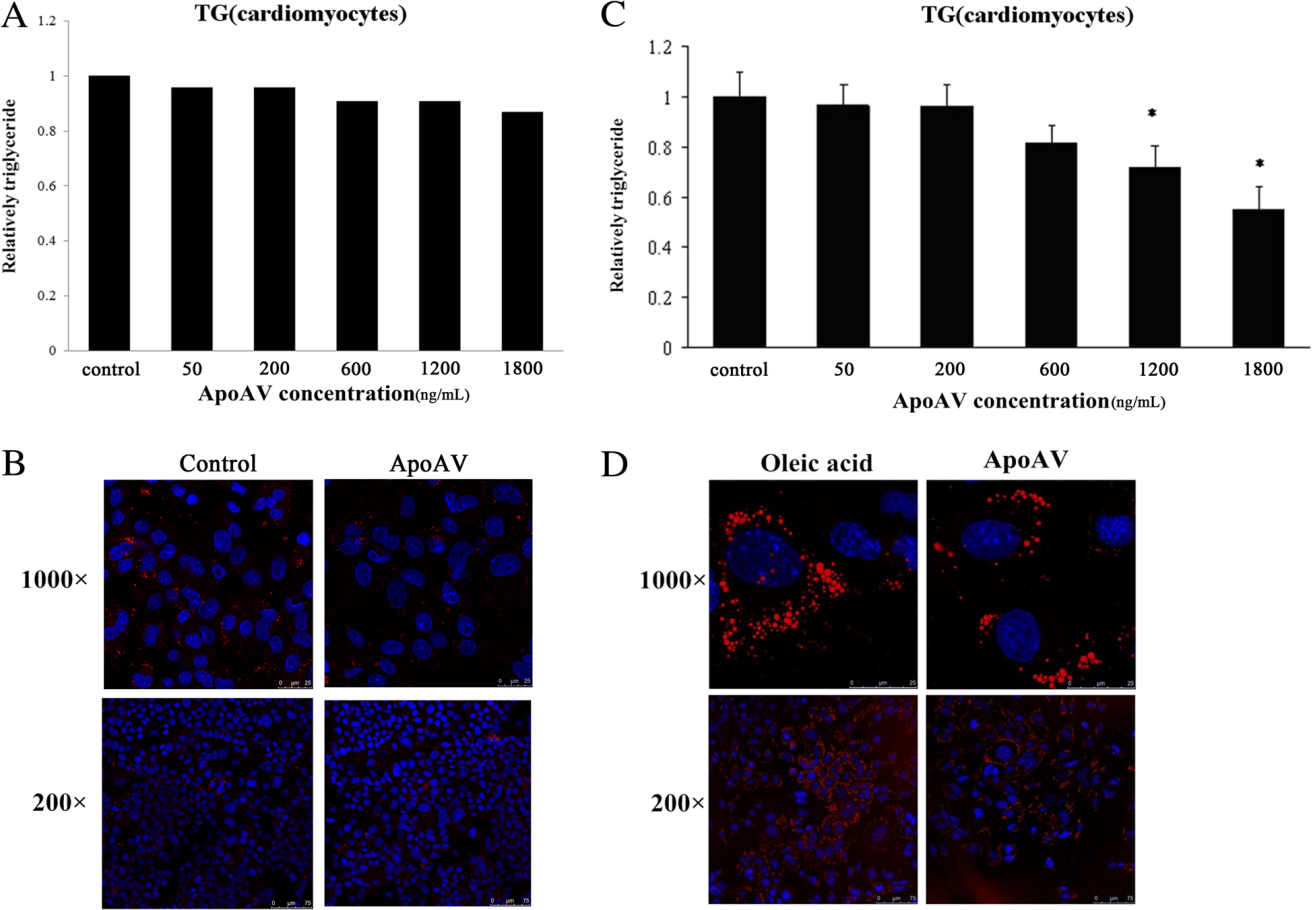
**a** Effect of apoA-V on the TG content in normal HL-1 cells. **b** Lipid droplet morphology visualized by fluorescence and confocal microscopy in normal HL-1 cells. **c** Effect of apoA-V on TG content in HL-1 cells with lipid accumulation. **d** Lipid droplet morphology visualized by fluorescence and confocal microscopy in normal HL-1 cells. Intervention with 1800 ng/ml of apoA-V significantly decreased the number of intracellular lipid droplets compared with the control group (without apoA-V intervention) but had no effect on the size of lipid droplets in HL-1 cells with lipid accumulation. (Confocal microscopy images were recorded at 1000× and 200× magnification, and scale bars are 25 and 75 μm, respectively; lipid droplets: red; nuclei: blue)

We next investigated the effect of apoA-V on TG content in HL-1 cells. In normal HL-1 cells, there was no significant change of intracellular TG levels in response to incubation with different concentrations of apoA-V (Fig. 5a). For HL-1 cells with lipid accumulation, results showed that intervention with 1200 and 1800 ng/ml apoA-V both significantly decreased intracellular TG levels by 28% and 45% (*p* < 0.05), respectively, while 50–600 ng/ml apoA-V had no significant effects on TG storage in HL-1 cells (Fig. 5c). This indicates that apoA-V can dose-dependently decrease TG content in HL-1 cells with lipid accumulation, while having no effects on normal HL-1 cells. To confirm these results, the effect of apoA-V on morphological changes of lipid droplets in HL-1 cells was studied using confocal microscopy. Images (Fig. 5d) revealed that intervention with 1800 ng/ml apoA-V significantly decreased the number of intracellular lipid droplets but had no effect on the size of lipid droplets in HL-1 cells with lipid accumulation. However, for normal HL-1 cells, microscope images revealed that intervention with 1800 ng/ml apoA-V had no effect on the size or number of lipid droplets (Fig. 5c).

### ApoA-V significantly decreases TG levels in mouse heart tissue

**Fig. 6:**
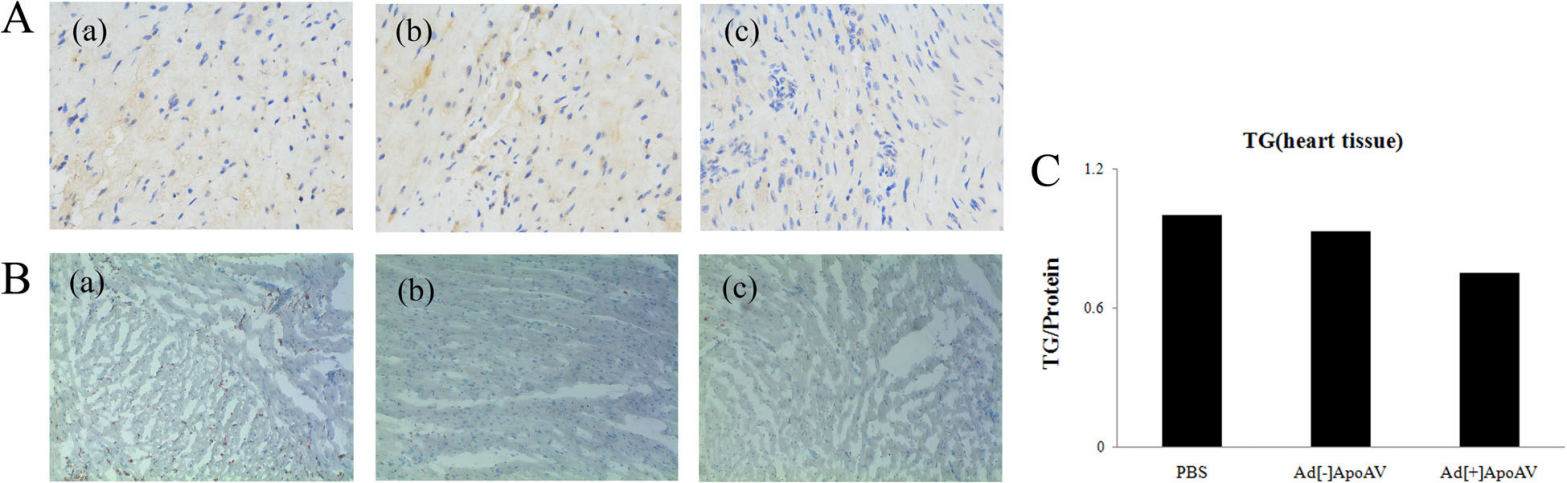
**A** Immunohistochemistry images of heart tissue from normal mice with (**A**-c) or without (**A**-b) the apoA-V adenovirus transfection. Controls were treated with PBS buffer (**A**-a). **B** Lipid accumulation in the heart tissue with (**B**-c) or without (**B**-b) apoA-V adenovirus transfection was examined by Oil Red Staining. Controls were treated with PBS buffer (**B**-a) (40× magnification). **C** TG content in heart tissue of normal mice with or without the apoA-V adenovirus transfection was examined with a triglyceride quantification kit; data are shown as the mean ± SE. *p < 0.05 vs. control

**Fig. 7:**
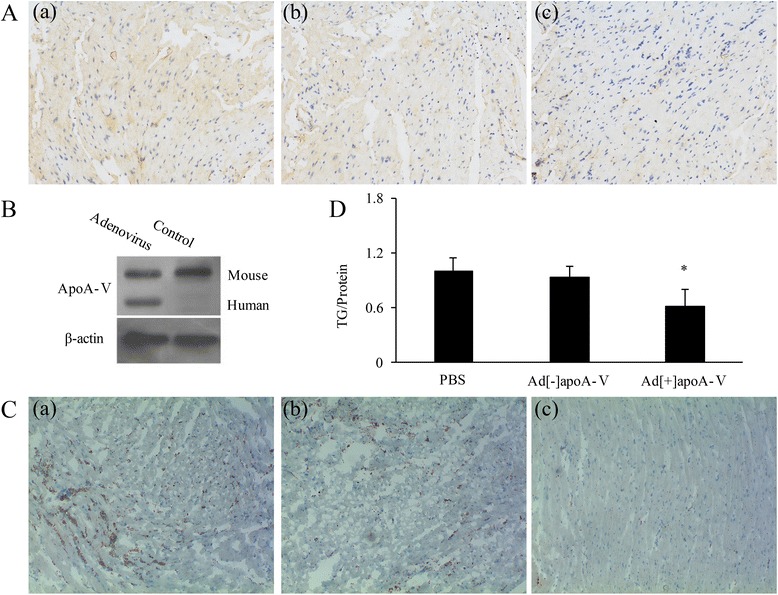
**A** Immunohistochemistry images of heart tissue from obese mice with (**A**-c) or without (**A**-b) apoA-V adenovirus transfection. Controls were treated with PBS buffer (**A**-a). **B** Western blot analysis of apoA-V levels in the heart tissue of control obese mice and those transfected with the apoA-V adenovirus. **C** Lipid accumulation in the heart tissue with (**C**)-c or without (**C**-b) apoA-V adenovirus transfection was examined by Oil Red Staining. Controls were treated with PBS buffer (**C**-a) (40× magnification). **D** TG content in the heart tissue of obese mice with or without apoA-V adenovirus transfection was examined with a Triglyceride Quantification Kit; data are shown as the mean ± SE. *p < 0.05 vs. control

We next investigated the effects of apoA-V on heart tissue in both normal and obese mice. Immunohistochemistry results showed that apoA-V expression was significantly increased in both normal and obese heart tissue transfected with apoA-V adenovirus (Figs. 6A, 7A). Western blot analysis also showed that apoA-V levels in mouse heart tissue transfected with apoA-V adenovirus were significantly increased (Fig. 7B). The reason for the heart tissue having two protein bands in mice with apoA-V transfection is because the apoA-V adenovirus comes from human, and the bands of human apoA-V adenovirus are displayed below the mouse bands. Although TG content in heart tissue of normal mice after transfection with apoA-V was decreased, this was not statistically significant (Fig. 6B and C). However, TG content was significantly decreased in the heart tissue of obese mice by 39% compared with that of the ad[−]apoA-V group (Fig. 7C and D).

### ApoA-V intervention alters cardiac expression of genes and proteins involved in fatty acid and triglyceride metabolism

**Fig. 8:**
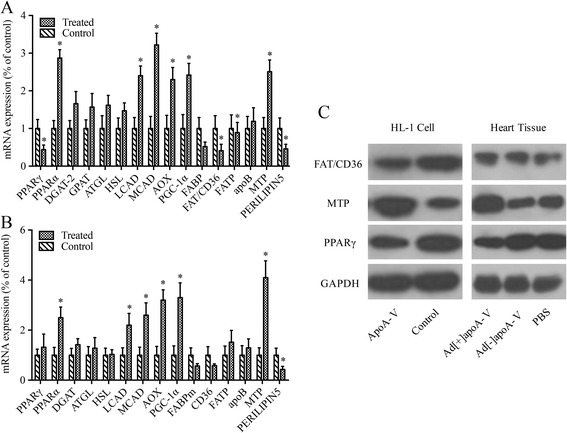
**a** mRNA expression levels involved in fatty acid and triglyceride metabolism in lipid-accumulated HL-1 cells with or without apoA-V treatment. **b** mRNA expression levels involved in fatty acid and triglyceride metabolism in the heart tissue of obese mice with or without the apoA-V transfection. **c** Western blot analysis of PPARα, PPARγ, MTP, and CD36 protein levels in HL-1 cells and mouse heart tissue

We investigated mRNA and protein expression levels of key enzymes and transcription factors involved in FA and TG metabolism in heart tissue both in vivo and in vitro. The results (Fig. 8a) showed that there is no significant change in mRNA expression levels of diacyl-glycerol-acyl-transferase 2 (DGAT-2), glycerol-3-phosphate acyltransferase (GPAT), hormone-sensitive lipase (HSL), adipose triglyceride lipase (ATGL), or fatty acid transport protein (FATP) in response to lipid accumulation in HL-1 cells when incubated with apoA-V, suggesting neither direct nor indirect effects of apoA-V on these enzymes or transcription factors. Remarkably, PPARα, medium-chain acyl-CoA oxidase (MCAD), long-chain acyl-CoA dehydrogenase (LCAD), acyl-CoA oxidase (AOX), PPARγ coactivator-1α (PGC-1α), and microsomal triglyceride transfer protein (MTP) mRNA expression levels were significantly increased by 2.8-, 3.2-, 2.4-, 2.3-, 2.4, and 2.5-fold, respectively. In contrast, mRNA expression of PPARγ, fatty acid translocase CD36 (FAT/CD36), fatty acid binding protein (FABP), and perilipin 5 (Plin5) were decreased by 56%, 59%, 48%, and 54%, respectively, by apoA-V intervention in cells (Fig. 8a), which was also supported by Western blot analyses (Fig. 8c).

Expression of the same genes was investigated in heart tissues of obese mice. mRNA expression levels related to FA oxidation and lipid secretion, such as MCAD, LCAD, AOX, PGC-1α, and MTP in obese mice transfected with apoA-V adenovirus were significantly increased by 2.6, 2.2, 3.2, 3.3, 3, and 4.1-fold, respectively. mRNA expression levels of PPARα were increased by 2.5-fold. In contrast to the in vitro results, apoA-V had no effects on gene expression involved in FA uptake, TG synthesis, or TG hydrolysis in obese mice transfected with apoA-V adenovirus compared to the control group (Fig. 8b). In agreement with the in vitro results, PPARγ expression in heart tissue was similar between apoA-V adenovirus mice and control mice. Although mRNA expression of TG hydrolysis genes (ATGL, HSL) was increased, it was not statistically significant (Fig. 4b). Western blot analyses showed similar results (Fig. 8c).

### ApoA-V intervention reduces the uptake of fatty acids and increases lipid secretion in cardiomyocytes

**Fig. 9:**
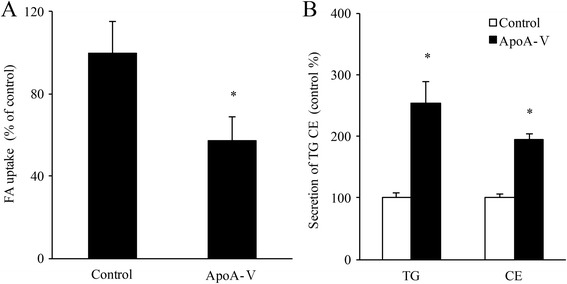
**a** Measurement of fatty acid uptake in HL-1 cells with or without 1800 ng/mL apoA-V intervention. **b** Measurement of TG and cholesterol ester secretion from HL-1 cells with or without 1800 ng/mL apoA-V intervention, *p < 0.05 vs. control

Compared to the cardiomyocytes without apoA-V intervention (control group), the levels of FA up take into cardiomyocytes were significantly reduced by 43% with apoA-V intervention using the isotope tracing method (*p* < 0.05) (Fig. 9a). However, apoA-V intervention significantly increased secretion of TG and cholesterol ester from HL-1 cells by 2.5-fold and 1.9-fold, respectively (*P* < 0.05) (Fig. 9b).

## Discussion

According to the World Health Organization (WHO), worldwide prevalence of obesity has doubled since 1980, with an estimated 1.5 billion adults having obesity in 2008 [21]. Obesity instigates accumulation of TG in cardiomyocytes [22]. TG accumulation and altered metabolism of FAs are associated with the development of myocardial contractile dysfunction and can lead to cardiomyocyte apoptosis [23]. Effectively reducing cardiac lipid accumulation caused by obesity is an international priority at present. Recently, clinical trials have demonstrated that apoA-V is an important determinant of plasma TG levels [1, 24]. In addition to regulating plasma TG levels, evidence indicates apoA-V plays a role in intracellular TG metabolism [25–27]. Thus, apoA-V is a potential therapeutic target for the modulation of plasma TG levels and obesity.

In this study, first we observed that apoA-V is taken up by HL-1 cells. Furthermore, apoA-V was previously shown to bind to receptors of the LDLR family, such as LRP1 [28]. Therefore, we established a cardiomyocyte culture model of LRP1 knockdown by transfecting normal and lipid-accumulated HL-1 cells with LRP1 siRNA. Our results showed that levels of apoA-V taken up into normal HL-1 cells were significantly decreased, while there was no significant difference in HL-1 cells with lipid accumulation. This suggests that endocytosis of apoA-V by cardiomyocytes is mediated, at least partly, by LDLR family members. LRP1 and VLDLR, two members of the LDLR family, are expressed in cardiomyocytes and involved in receptor-mediated endocytosis of lipoprotein particles [29, 30]. Experiments using surface plasmon resonance showed specific binding of apoA-V to LRP1, although VLDLR is also abundantly expressed in heart tissue, and its expression is increased in obese mice [31]. Whether apoA-V binds to VLDLR has not been demonstrated and remains to be studied. We speculate that apoA-V may also bind to VLDLR in cardiomyocytes. This is why in HL-1 cells with lipid accumulation, the rates of apoA-V taken up into cells have no statistical significance when compared with cells without apoA-V siRNA transfection.

Lipid droplets are key components of neutral lipids (TG or cholesteryl ester) and function as dynamic lipid storage depots [32]. They can be found in nearly all cell types, including cardiomyocytes. In the present study, the results showed that normal HL-1 cells rarely contain lipid droplets, but when HL-1 cells were co-incubated with oleic acid, the amount of lipid droplets in them increased significantly. Furthermore, the confocal microscopy data suggest that apoA-V associates with cytosolic lipid droplets, which is consistent with previous observations that apoA-V targeted cytosolic lipid droplets in hepatoma cell lines transfected with apoA-V or in human adipocytes [3]. Given the findings that apoA-V associated with lipid droplets in cardiomyocytes, it is reasonable to hypothesize that apoA-V can function to regulate intracellular TG metabolism. In the present study, we found that apoA-V significantly reduces intracellular TG levels in HL-1 cells with lipid accumulation. The same effects were observed in obese mice transfected with apoA-V adenovirus compared with control obese mice. However, there were no changes of TG content in normal HL-1 cells and mice with apoA-V intervention. The reasons for this difference may be that TG levels are too low in normal cells and heart tissue, so apoA-V intervention would not lower them. Given that TG accumulation and altered metabolism of FAs due to obesity are associated with the development of myocardial contractile dysfunction and can lead to cardiomyocyte apoptosis [33], it is conceivable that apoA-V plays a key role in heart failure protection via reducing lipid accumulation.

To further explore the underlying mechanisms through which apoA-V directly or indirectly influences lipid metabolism in cardiomyocytes, we measured mRNA expression levels of key enzymes and transcription factors involved in heart lipid metabolism both in vivo and in vitro. PPARα plays an important role in FA metabolism and is abundantly expressed in heart muscle cells [34]. In our study, PPARα was increased in both HL-1 cells and obese mice when supplemented with apoA-V. Moreover, it has been demonstrated that some lipid-derived molecules, including long-chain FAs, eicosanoids, and leukotriene B4 activate PPARα [35]. Differences between previous and recent studies may be due to the variations in cell types. Likewise, PPARα mRNA expression has been demonstrated to be activated by FAs both in vivo and in vitro, not by the direct effect of apoA-V. PPARγ is adipose-enriched, controls the expression of genes involved in FAs storage and adipogenesis, and is expressed at low levels in extra-adipose tissues including the skeletal muscle, pancreatic β-cell, and heart tissue. Recently, Luo et al. found that the transcription and protein levels of important proteins in FA uptake and oxidation were reduced in PPARγ deficient hearts [36]. Moreover, Ress et al. found that PPARγ mRNA expression levels were significantly increased in apoA-V siRNA transfected HepG2 cells when compared with control cells transfected with non-silencing siRNA [5]. Based on these studies and our results, we demonstrated that apoA-V decreases PPARγ expression and lipid accumulation in cardiomyocytes. Furthermore, PGC-1 has recently emerged as a crucial player in the regulation of myocardial metabolism. Consistent with its functional interaction with PPARs, PGC-1 activates the expression of genes involved in FA uptake and oxidation when overexpressed in cardiomyocytes [37]. In our study where PGC-1α expression was increased, mRNA levels of LCAD, MCAD, and AOX increased both in vivo and in vitro, demonstrating that mitochondrial FAs β-oxidation was increased in cardiomyocytes in response to apoA-V intervention. Enzymes of FA β-oxidation, such as MCAD and LCAD, are also under a high degree of transcriptional control and have been proven to be regulated by PPARα [38]. Thus, it is reasonable that FAs oxidation was increased, while PPARα mRNA expression was also increased. Furthermore, recent studies on rats [39], mice [40], and humans [41] have shown that cardiac FA oxidation rates are actually elevated by obesity, insulin resistance, and type 2 diabetes. It was concluded that apoA-V increases FA oxidation in heart tissue with lipid accumulation. However, whether apoA-V intervention can increase myocardial apoptosis after these effects has not been studied in detail.

Genes involved in FA uptake were down regulated with apoA-V intervention in vitro (CD36, FABPm), but this result was not reproducible in obese mice transfected with apoA-V adenovirus. Although the heart has the ability to store FAs, it has a limited capacity to maintain its high metabolic demand on endogenous sources alone, relying on continuous uptake from blood [42]. Both in vitro and in vivo studies have shown that FAT/CD36-mediated FA transport in the heart accounts for 60–80% of the FAs taken up by cardiomyocytes [43]. We found that mRNA levels of CD36 and FABP were decreased in HL-1 cells supplemented with apoA-V when compared with controls and that the rates of FAs taken up into cardiomyocytes were reduced when measured with [^14^C]-oleic acid. However, there was significant change in mRNA levels of CD36, FABP, and FATP in vivo. This may be due to the discrepancy between in vivo and in vitro interpreted as cell- and organ-specific effects of apoA-V intervention. In addition, we found that there is no significant change in mRNA expression of DGAT, GPAM, ATGL, and HSL in vivo and in vitro by apoA-V intervention, indicating that apoA-V did not influence cardiac TG synthesis and hydrolysis.

The heart is a lipoprotein-secreting organ in both mice and humans, similar to the liver and intestines. Production of lipoproteins in the heart is mediated by apoB and MTP and unregulated by myocardial MTP following fasting and high-fat diet-induced obesity [44]. This suggests that MTP activity and cardiac lipoprotein secretions are activated as a protective mechanism to promote reversal of TG transport under conditions that lead to increased myocardial FA uptake and TG accumulation. In our study, apoA-V intervention unregulated MTP mRNA expression both in vivo and in vitro, and the levels of TG and cholesterol ester secreted from myocardial cells were increased by treated with [^14^C]-oleic acid. This demonstrates that apoA-V further increases lipoprotein secretion in cardiomyocytes and protect from excess lipid deposition during diet-induced obesity. More recently, Plin5 was found to be highly present on lipid droplets of oxidative tissues including cardiac, skeletal muscle, and liver tissue [45]. Ectopic expression of Plin5 increased cellular TG levels and reduced FA oxidation, indicating a role for Plin5 in energy catabolism. Here, we showed that Plin5 mRNA expression was decreased in vivo and in vitro during apoA-V administration, suggesting that lipid accumulation in cardiomyocytes reduced by apoA-V intervention may also be due to the down regulation of Plin5, promoting TG hydrolysis.

## Conclusion

In summary, we found that apoA-V is taken up by cardiomyocytes, at least partly via binding to members of LDLR family, and can decrease TG content and lipid accumulation in HL-1 cells with lipid accumulation and heart tissue of obese mice. ApoA-V promotes FA oxidation and increases lipid secretion from cardiomyocytes, decreasing the levels of FAs taken up into cardiomyocytes. These data indicate that apoA-V acts as a novel regulator to modulate TG storage in cardiomyocytes. These findings suggest that apoA-V could be a potential therapeutic target for the treatment of myocardial lipid deposition, and further investigation is thus warranted.

## Abbreviations

ApoA-V: Apolipoprotein A-V
FAs: Fatty acids
HFD: High fat diet
LDLR: Low density lipoprotein receptor
LRP1: LDLR-related protein 1
PPAR: Peroxisome proliferator-activated receptor
TG: Triglyceride

## References

[1] Pennacchio LA, Olivier M, Hubacek JA, Cohen JC, Cox DR, Fruchart JC, Krauss RM, Rubin EM (2001). An apolipoprotein influencing triglycerides in humans and mice revealed by comparative sequencing. Science.

[2] van der Vliet HN, Schaap FG, Levels JH, Ottenhoff R, Looije N, Wesseling JG, Groen AK, Chamuleau RA (2002). Adenoviral overexpression of apolipoprotein A-V reduces serum levels of triglycerides and cholesterol in mice. Biochem Biophys Res Commun.

[3] Zheng XY, Zhao SP, Yu BL, Wu CL, Liu L (2012). Apolipoprotein A5 internalized by human adipocytes modulates cellular triglyceride content. Biol Chem.

[4] Zheng XY, Yu BL, Xie YF, Zhao SP, Wu CL (2017). Apolipoprotein A5 regulates intracellular triglyceride metabolism in adipocytes. Mol Med Rep.

[5] Ress C, Moschen AR, Sausgruber N, Tschoner A, Graziadei I, Weiss H, Schgoer W, Ebenbichler CF, Konrad RJ, Patsch JR (2011). The role of apolipoprotein A5 in non-alcoholic fatty liver disease. Gut.

[6] Shu X, Nelbach L, Ryan RO, Forte TM (1801). Apolipoprotein A-V associates with intrahepatic lipid droplets and influences triglyceride accumulation. Biochim Biophys Acta.

[7] Hubert HB, Feinleib M, Mcnamara PM, Castelli WP (1983). Obesity as an independent risk factor for cardiovascular disease- a 26-year follow-up of participants in the Framingham heart-study. Circulation.

[8] Mokdad AH, Bowman BA, Ford ES, Vinicor F, Marks JS, Koplan JP (2001). The continuing epidemics of obesity and diabetes in the United States. JAMA-J Am Med Assoc.

[9] Wilson PWF, D'Agostino RB, Sullivan L, Parise H, Kannel WB (2002). Overweight and obesity as determinants of cardiovascular risk - the Framingham experience. JAMA Inter Med.

[10] Kenchaiah S, Evans JC, Levy D, Wilson PWF, Benjamin EJ, Larson MG, Kannel WB, Vasan RS (2002). Obesity and the risk of heart failure. New Engl J Med.

[11] Ng ACT, Delgado V, Bertini M, van der Meer RW, Rijzewijk LJ, Ewe SH, Siebelink HM, Smit JWA, Diamant M, Romijn JA (2010). Myocardial steatosis and biventricular strain and strain rate imaging in patients with type 2 diabetes mellitus. Circulation.

[12] Ouwens DM, Boer C, Fodor M, de Galan P, Heine RJ, Maassen JA, Diamant M (2005). Cardiac dysfunction induced by high-fat diet is associated with altered myocardial insulin signalling in rats. Diabetologia.

[13] Tao NJ, Gao GP, Parr M, Johnston J, Baradet T, Wilson JM, Barsoum J, Fawell SE (2001). Sequestration of adenoviral vector by Kupffer cells leads to a nonlinear dose response of transduction in liver. Mol Ther.

[14] Lamberts RR, Van Rijen MHP, Sipkema P, Fransen P, Sys SU, Westerhof N (2002). Coronary perfusion and muscle lengthening increase cardiac contraction: different stretch-triggered mechanisms. Am J Physiol Heart Circ Physiol.

[15] Claycomb WC, Lanson NA, Stallworth BS, Egeland DB, Delcarpio JB, Bahinski A, Izzo NJ (1998). HL-1 cells: a cardiac muscle cell line that contracts and retains phenotypic characteristics of the adult cardiomyocyte. Proc Natl Acad Sci U S A.

[16] Beckstead JA, Oda MN, Martin DDO, Forte TM, Bielicki JK, Berger T, Luty R, Kay CM, Ryan RO (2003). Structure-function studies of human apolipoprotein A-V: a regulator of plasma lipid homeostasis. Biochemistry.

[17] Braun NA, Mohler PJ, Weisgraber KH, Hasty AH, Linton MF, Yancey PG, Yan RS, Fazio S, Swift LL (2006). Intracellular trafficking of recycling apolipoprotein E in Chinese hamster ovary cells. J Lipid Res.

[18] Luiken JJFP, vanNieuwenhoven FA, America G, vanderVusse GJ, Uptake GJFC (1997). Metabolism of palmitate by isolated cardiac myocytes from adult rats: involvement of sarcolemmal proteins. J Lipid Res.

[19] Bligh EG, Dyer WJ (1959). A rapid method of Total lipid extraction and purification. Can J Biochem Physiol.

[20] Guardiola M, Alvaro A, Vallve JC, Rosales R, Sola R, Girona J, Serra N, Duran P, Esteve E, Masana L, Ribalta J (2012). APOA5 gene expression in the human intestinal tissue and its response to in vitro exposure to fatty acid and fibrate. Nutr Metab Cardiovas.

[21] Chan JCN, Malik V, Jia WP, Kadowaki T, Yajnik CS, Yoon KH, Hu FB (2009). Diabetes in Asia epidemiology, risk factors, and pathophysiology. JAMA.

[22] McGavock JM, Lingvay I, Zib I, Tillery T, Salas N, Unger R, Levine BD, Raskin P, Victor RG, Szczepaniak LS (2007). Cardiac steatosis in diabetes mellitus: a 1H-magnetic resonance spectroscopy study. Circulation.

[23] Christoffersen C, Bollano E, Lindegaard ML, Bartels ED, Goetze JP, Andersen CB, Nielsen LB (2003). Cardiac lipid accumulation associated with diastolic dysfunction in obese mice. Endocrinology.

[24] van der Vliet HN, Sammels MG, Leegwater AC, Levels JH, Reitsma PH, Boers W, Chamuleau RA (2001). Apolipoprotein A-V: a novel apolipoprotein associated with an early phase of liver regeneration. J Biol Chem.

[25] Zhao SP, Li R, Dai W, Yu BL, Chen LZ, Huang XS (2017). Xuezhikang contributes to greater triglyceride reduction than simvastatin in hypertriglyceridemia rats by up-regulating apolipoprotein A5 via the PPARalpha signaling pathway. PLoS One.

[26] Wu CL, Zhao SP, Yu BL (2015). Intracellular role of exchangeable apolipoproteins in energy homeostasis, obesity and non-alcoholic fatty liver disease. Biol Rev Camb Philos Soc.

[27] Luo F, Guo Y, Ruan GY, Peng R, Li XP (2017). Estrogen lowers triglyceride via regulating hepatic APOA5 expression. Lipids Health Dis.

[28] Nilsson SK, Lookene A, Beckstead JA, Gliemann J, Ryan RO, Olivecrona G (2007). Apolipoprotein A-V interaction with members of the low density lipoprotein receptor gene family. Biochemistry.

[29] Castellano J, Aledo R, Sendra J, Costales P, Juan-Babot O, Badimon L, Llorente-Cortes V (2011). Hypoxia stimulates low-density lipoprotein receptor-related protein-1 expression through hypoxia-inducible factor-1alpha in human vascular smooth muscle cells. Arterioscler Thromb Vasc Biol.

[30] Cal R, Castellano J, Revuelta-Lopez E, Aledo R, Barriga M, Farre J, Vilahur G, Nasarre L, Hove-Madsen L, Badimon L, Llorente-Cortes V (2012). Low-density lipoprotein receptor-related protein 1 mediates hypoxia-induced very low density lipoprotein-cholesteryl ester uptake and accumulation in cardiomyocytes. Cardiovasc Res.

[31] Hauton D, Caldwell GM (1821). Cardiac lipoprotein lipase activity in the hypertrophied heart may be regulated by fatty acid flux. BBA-Mol CellL Biol L.

[32] Brasaemle DL (2007). The perilipin family of structural lipid droplet proteins: stabilization of lipid droplets and control of lipolysis. J Lipid Res.

[33] Wende AR, Abel ED (1801). Lipotoxicity in the heart. Biochim Biophys Acta.

[34] Barger PM, Brandt JM, Leone TC, Weinheimer CJ, Kelly DP (2000). Deactivation of peroxisome proliferator-activated receptor-alpha during cardiac hypertrophic growth. J Clin Invest.

[35] Devchand PR, Keller H, Peters JM, Vazquez M, Gonzalez FJ, Wahli W (1996). The PPARalpha-leukotriene B4 pathway to inflammation control. Nature.

[36] Luo J, Wu S, Liu J, Li Y, Yang H, Kim T, Zhelyabovska O, Ding G, Zhou Y, Yang Y, Yang Q (2010). Conditional PPARgamma knockout from cardiomyocytes of adult mice impairs myocardial fatty acid utilization and cardiac function. Am J Transl Res.

[37] Lehman JJ, Barger PM, Kovacs A, Saffitz JE, Medeiros DM, Kelly DP (2000). Peroxisome proliferator-activated receptor gamma coactivator-1 promotes cardiac mitochondrial biogenesis. J Clin Invest.

[38] Watanabe K, Fujii H, Takahashi T, Kodama M, Aizawa Y, Ohta Y, Ono T, Hasegawa G, Naito M, Nakajima T (2000). Constitutive regulation of cardiac fatty acid metabolism through peroxisome proliferator-activated receptor alpha associated with age-dependent cardiac toxicity. J Biol Chem.

[39] Aasum E, Hafstad AD, Severson DL, Larsen TS (2003). Age-dependent changes in metabolism, contractile function, and ischemic sensitivity in hearts from db/db mice. Diabetes.

[40] Wang PP, Lloyd SG, Zeng HD, Bonen A, Chatham JC (2005). Impact of altered substrate utilization on cardiac function in isolated hearts from Zucker diabetic fatty rats. Am J Physiol Heart Circ Physiol.

[41] Peterson LR, Herrero P, Schechtman KB, Racette SB, Waggoner AD, Kisrieva-Ware Z, Dence C, Klein S, Marsala J, Meyer T, Gropler RJ (2004). Effect of obesity and insulin resistance on myocardial substrate metabolism and efficiency in young women. Circulation.

[42] Vandervusse GJ, Glatz JFC, Stam HCG, Reneman RS (1992). Fatty-acid homeostasis in the normoxic and ischemic heart. Physiol Rev.

[43] Carley AN, Kleinfeld AM (2011). Fatty acid (FFA) transport in cardiomyocytes revealed by imaging unbound FFA is mediated by an FFA pump modulated by the CD36 protein. J Biol Chem.

[44] Nielsen LB, Bartels ED, Bollano E (2002). Overexpression of apolipoprotein B in the heart impedes cardiac triglyceride accumulation and development of cardiac dysfunction in diabetic mice. J Biol Chem.

[45] Wang H, Sztalryd C (2011). Oxidative tissue: perilipin 5 links storage with the furnace. Trends Endocrinol Metab.

